# Tamm-cavity terahertz detector

**DOI:** 10.1038/s41467-024-49759-z

**Published:** 2024-07-02

**Authors:** Xuecou Tu, Yichen Zhang, Shuyu Zhou, Wenjing Tang, Xu Yan, Yunjie Rui, Wohu Wang, Bingnan Yan, Chen Zhang, Ziyao Ye, Hongkai Shi, Runfeng Su, Chao Wan, Daxing Dong, Ruiying Xu, Qing-Yuan Zhao, La-Bao Zhang, Xiao-Qing Jia, Huabing Wang, Lin Kang, Jian Chen, Peiheng Wu

**Affiliations:** 1 Research Institute of Superconductor Electronics (RISE), School of Electronic Science and Engineering, Nanjing University, Nanjing, Jiangsu 210023 China; 2 Hefei National Laboratory, Hefei, 230088 China; 3 Purple Mountain Laboratories, Nanjing, Jiangsu 211111 China; 4 Department of Applied Physics, Nanjing University of Aeronautics and Astronautics, Nanjing, 210016 China; 5 Nanjing Electronic Devices Institute, Nanjing, 210016 China

**Keywords:** Photonic devices, Terahertz optics

## Abstract

Efficiently fabricating a cavity that can achieve strong interactions between terahertz waves and matter would allow researchers to exploit the intrinsic properties due to the long wavelength in the terahertz waveband. Here we show a terahertz detector embedded in a Tamm cavity with a record Q value of 1017 and a bandwidth of only 469 MHz for direct detection. The Tamm-cavity detector is formed by embedding a substrate with an Nb_5_N_6_ microbolometer detector between an Si/air distributed Bragg reflector (DBR) and a metal reflector. The resonant frequency can be controlled by adjusting the thickness of the substrate layer. The detector and DBR are fabricated separately, and a large pixel-array detector can be realized by a very simple assembly process. This versatile cavity structure can be used as a platform for preparing high-performance terahertz devices and opening up the study of the strong interactions between terahertz waves and matter.

## Abstract (web summary)

Here the authors report a terahertz detector with a Q value of 1017, embedded in a Tamm cavity and offers a 469 MHz bandwidth. It features an Nb5N6 microbolometer in an Si/air DBR and metal reflector, with tunable resonant frequency via substrate layer thickness.

## Introduction

In recent years, thanks to the development of terahertz sources^1–4^, detectors^5–10^, modulators^11–13^, and other devices^14,15^, many remarkable results in imaging^16–19^, molecular gas detection^20,21^, and communication^22–25^ in terahertz science and technology^26^ have been achieved. One of the most important scientific issues for the development of these devices is how to enhance the strong interactions between these devices and terahertz signals to achieve efficient coupled input or output^27–34^. Optical resonators and nanocavities, such as Fabry–Pérot (FP) interferometers^35,36^, microcavities^37–40^, photonic crystals^41,42^, and planar resonators^43,44^, are powerful tools for producing strong interactions^45^. In particular, enhanced structures based on a Tamm cavity^46–50^ with a built-in distributed Bragg reflector (DBR) are commonly used in photodetectors^51–54^ and lasers^55–57^. Since Tamm cavities in the optical band based on a metal DBR were first proposed by ref. ^47^, they have had an important role in enhancing the interaction between material and light to realize high *Q* and tunable devices^58–60^. These excellent properties are necessary for terahertz-band devices. However, the functional components integrated with the DBR in the terahertz spectral range have rarely been reported in the literature. The main difficulty is that the smallest planar features are of the order of λ/4*n*_r_ ≈ 10 μm, where *n*_r_ is the refractive index of the dielectric. Terahertz wavelengths are in the range 10 to 1000 μm, so depositing thin films of an optical dielectric, which is commonly used for optical devices, cannot be used to construct microcavities, which is necessary for the DBR structures used in terahertz devices.

Note that the features of terahertz microcavities are of the order of the thickness of the substrates of the terahertz devices, so researchers have also begun to use the substrates as FP cavities when constructing electromagnetic confinement devices^61–63^. To obtain a tunable terahertz device, the length of the cavity can be changed with an electronically controlled displacement platform or a microelectromechanical system (MEMS) with micrometer precision^64,65^. These FP cavities have greatly facilitated the development of terahertz components that are continuously frequency adjustable and have a wideband response^66,67^. Obviously, the performance of these devices can be further improved if the Tamm cavity is used as in optical wavebands^68,69^. An optical DBR cavity composed of multiple layers of Si and air was fabricated by an ingenious and complex process. It has a very high refractive index contrast and very high *Q* value. This device has been used in various high-performance lasers^70–72^. Obviously, the difficulties in depositing dielectric thin films and lateral etching are hard to address at the micro-scale in the terahertz band. Therefore, a detector or source integrated with a Tamm cavity in the terahertz band has not been reported experimentally.

Here we propose a terahertz detector embedded in a Tamm cavity, which consists of a DBR with silicon/air layers, a microbolometer detector deposited on silicon substrate, and a reflective mirror. In physics, this structure can be seen as a hybrid Tamm cavity formed by inserting a dielectric layer into a pure Tamm cavity, and the terahertz detector is prepared on this dielectric layer. The air and silicon dielectric layers are formed by deep silicon etching in the same high-resistivity float-zone (HRFZ) Si wafer chip. The silicon chip containing the air cavity is stacked and bonded with a photoresist to form a DBR with multiple Si/air layers. It is bonded to the detector chip containing a microbolometer detector and a substrate layer to form the hybrid Tamm cavity. The resonant modes of the detector can be tuned by controlling the thickness of the substrate of the microbolometer detector chip. The DBR and detector are fabricated separately, which reduces the complexity of design and fabrication. Large-scale fabrication can be achieved by simple MEMS process and bonding assembly, which is also compatible with other terahertz devices. This approach overcomes the drawbacks that millimeter-scale multilayers are hard to control precisely and integrate with terahertz devices^73^.

We demonstrate experimentally that the Tamm cavity coupled detector has high *Q* and very narrowband optical responsivity in the terahertz band. It provides a general operating platform for other devices that need enhanced interactions between matter and a terahertz wave. In particular, it can be used to study the electronics and optoelectronics of 2D materials^74–77^ or to fabricate terahertz lasers, terahertz detectors, and other high-performance functional devices.

## Results

### Device design

**Fig. 1:**
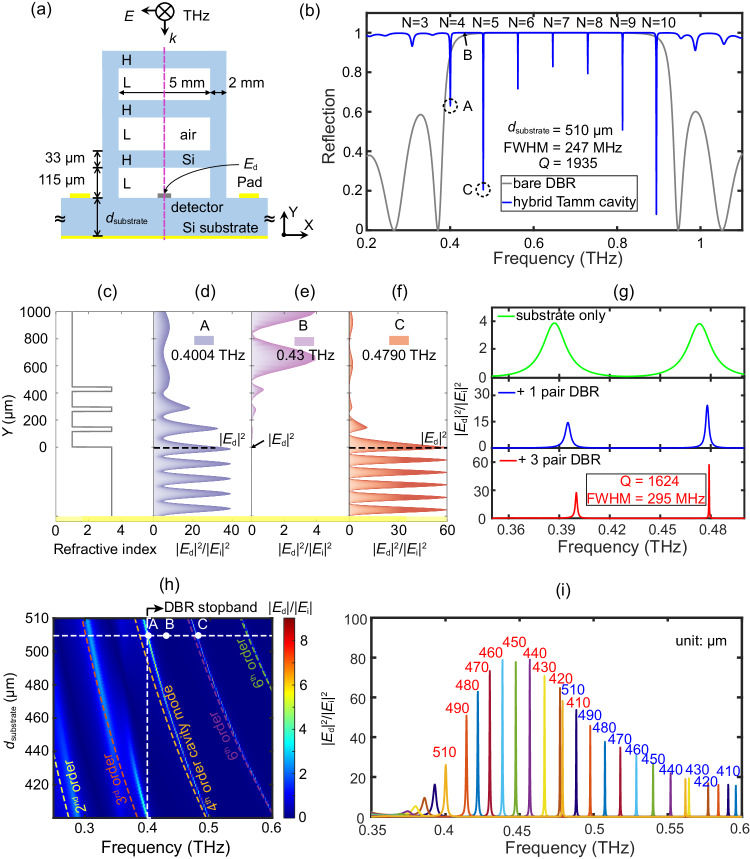
Schematics of the hybrid Tamm-cavity detector operating in the terahertz band and its resonance characteristics. **a** Schematic diagram of the hybrid Tamm-cavity detector. Silicon is shown in gray, air in white, and the mirror back on the substrate in yellow. **b** Reflectance spectra of a bare three-layer DBR and a hybrid Tamm cavity at *d*_substrate_ = 510 μm. The different extremum points correspond to the cavity’s resonant modes in Eq. (2). **c** Spatial distribution of the refractive index of the multilayer dielectrics in the hybrid Tamm cavity in the vertical direction. The yellow region represents the position of the metal mirror at the back of the detector chip with *d*_substrate_ = 510 μm. **d**–**f** Spatial distributions of the enhancement factor of the electric field intensity (|*E*|^2^/|E_i_|^2^) along the vertical purple dashed line in (**a**) at 0.4004 THz [*A* in (**b**)], 0.4300 THz [*B* in (**b**)], and 0.4790 THz [*C* in (**b**)]. The black dashed lines indicate the electric field intensity |*E*_d_|^2^ at the detector (*Y* = *d*_substrate_ = 510 μm). **g** Electric field enhancement factor (|*E*_d_|^2^/|E_i_|^2^) at the detector with zero, one, or three Si/air layers with *d*_substrate_ = 510 μm. **h** Relation between the electric field (|*E*_d_|) and substrate thickness of the detector chip (*d*_substrate_) for a hybrid Tamm cavity with three Si/air layers in the range 0.25–0.6 THz. The five white dashed lines were calculated by Eq. (2) and indicate the resonant modes of the cavity with *N* = 2, 3, 4, 5, or 6, respectively. The horizontal white line indicates the resonance characteristics of the hybrid Tamm cavity at *d*_substrate_ = 510 μm. The dots labeled *A*, *B*, and *C* correspond to the cases illustrated in (**b**, **d**, **e**), and (**f**). The band gap of DBR is also marked with vertical white dashed lines on the graph. **i** Spectral characteristics of the electric field intensity |*E*_d_|^2^ in a hybrid Tamm cavity with different thicknesses of the substrates of the detector chips.

As shown in Fig. 1a, the hybrid Tamm cavity structure is formed by sandwiching a HRFZ-Si substrate between a DBR with three Si/air layers, a terahertz detector, and a reflective metal mirror. The detector, which is a Nb_5_N_6_ microbolometer, is embedded in this structure through being deposited on the HRFZ-Si substrate that controls the electric field intensity at the detector. The detector can, in principle, be any electric field detector. It acts as a near-field terahertz probe^78^. Here, we used a Nb_5_N_6_ microbolometer whose voltage responsivity is proportional to the electric field intensity. The key to designing a hybrid Tamm cavity detector is realizing the DBR in the terahertz band and ensuring it is compatible with the detector integration process. A significant advantage of the hybrid Tamm cavity is that the detector chip and the DBR can be prepared separately, making the design and fabrication simple. The HRFZ-Si is, undoubtedly, the best choice for constructing this DBR due to the small losses in the terahertz band^79,80^ and its compatibility with standard silicon MEMS processes. The air layer can be obtained by Si etching using a square opening in the same chip. More importantly, DBR made of HRFZ-Si and air has a large refractive index contrast (*n*_si_ = 3.4147 and *n*_air_ = 1), resulting in a wide transmission stopband. The thickness of the substrate of the detector chip is determined primarily according to the desired detection band, after which the thicknesses of the silicon and air layers in the DBR are optimized. To detect electromagnetic waves around 0.65 THz, according to the resonant conditions in the cavity modes, the thickness of the detector chip was 510 μm, which can support this resonant mode. According to design theory for a DBR, we let *n*_H_*d*_H_ = *n*_L_*d*_L_ = λ/4, where λ is the target resonant wavelength, *n*_H_ = 3.4147 is the refractive index of silicon, *d*_H_ = 33 μm is the thickness of the silicon layer, *n*_L_ = 1 is the refractive index of air, and *d*_L_ = 115 μm is the thickness of the air layer.

By using the electromagnetic wave transfer-matrix method (TMM) of multilayer media (Supplementary Note S1), the reflection spectrum of a DBR with three Si/air layers is calculated, as shown by the gray solid line in Fig. 1b. The DBR reflects up to 100% in the band from 0.5 to 0.8 THz. A spectrally wide stop band filter is realized just by three Si/air layers, which benefits from the high refractive index contrast between HRFZ-Si and air. The reduced period number also reduces fabrication and micro-assembly errors.

Figure 1c shows the spatial distribution of the refractive index of each layer of material of the final hybrid Tamm cavity shown in Fig. 1a. The Tamm modes^46,47^ occur in this hybrid Tamm cavity under certain conditions for a one-dimensional photonic crystal. The detector substrate acts as a dielectric resonant cavity in this hybrid Tamm cavity, and the phase change is described by $4\pi {n}_{{{{{{\rm{Si}}}}}}}{d}_{{{{{{\rm{cavity}}}}}}}/\lambda$ in a round trip. The thickness of the substrate should satisfy the following condition if Tamm resonance occurs in the entire hybrid Tamm cavity^47,56^:

1 $${r}_{{{{{{\rm{DBR}}}}}}}{r}_{{{{{{\rm{Au}}}}}}}{e}^{{{{{{\rm{i}}}}}}(2{n}_{{{{{{\rm{Si}}}}}}}{d}_{{{{{{\rm{cavity}}}}}}})}=1$$

The phase condition at the resonant point of the hybrid Tamm cavity fully conforms to the conditions for optical Tamm states (Supplementary Note S2), indicating that there is also an “optical band-like” Tamm mode in the terahertz band. This is special and different because it not only satisfies the conditions for a Tamm state but also maximizes the electric field at the position of the detector [i.e., *E*_d_ in Fig. 1a]. The thickness of the detector substrate in the cavity must also satisfy the following condition:

2 $${d}_{{{{{{\rm{substrate}}}}}}}=\frac{(2N+1)\lambda }{4{n}_{{{{{{\rm{Si}}}}}}}}$$

where *N* is the resonant mode of the FP cavity of the detector substrate. This is exactly the condition for the enhancement of the coherence of the electric field in the detector substrate layer mainly, that is, the thickness of the detector substrate determines the resonant frequency of the entire hybrid Tamm cavity in the band gap of DBR. Thus, the resonant modes of this hybrid Tamm cavity are mainly determined by the thickness of substrate layer (i.e., *d*_substrate_, the thickness of the microbolometer substrate here)^56^. This finding is verified by the following calculated results.

The blue line in Fig. 1b is the reflection coefficient of the entire hybrid Tamm-cavity. with *d*_substrate_ = 510 μm calculated by the TMM (Supplementary Note S1). Multiple resonant extremum points (Tamm modes) were formed due to the attachment of the detector chip to the substrate layer. *N* is the resonant mode, as determined by Eq. (2). At 0.4790 THz, more than 90% of the energy is confined to the detector substrate and the *Q* value is up to 1935. The full width at half maximum (FWHM) is only 247 MHz. The electric field distributions are calculated at the center of the hybrid Tamm cavity. [the purple dashed line in the direction of the *y*-axis in Fig. 1a] for points *A* (0.4004 THz), *B* (0.4300 THz), and *C* (0.4790 THz), as shown in Fig. 1d–f. The electric field intensity at the detector |*E*_d_|^2^ is shown as the black dashed line. In the simulation, the electric field intensity of the incident terahertz plane wave |*E*_i_| is set as 1, and |*E*|^2^/|*E*_i_|^2^ represents the enhancement factor of the electric field intensity at the purple dashed line in Fig. 1a. At point *A*, since the reflection coefficient is 0.6 and the electric field at the detector was not at a node of the standing wave, the enhancement factor was only 32. At point *B*, the electric field intensity at the detector was 0 due to total reflection of the incident terahertz wave. At point *C*, the reflection coefficient was only 0.2 and the electric field at the detector was at a node of a standing wave in the entire hybrid Tamm cavity, so the enhancement factor was a maximum of up to 57. The electromagnetic field oscillated in the substrate layer, and the energy was confined to the substrate layer and eventually absorbed by the detector, greatly enhancing the response sensitivity of the detector. This kind of hybrid Tamm cavity significantly enhances the interaction between terahertz waves and the sensor. There is a significant difference in the electric field intensity at different locations, so it is important to precisely control the thickness of each layer of the media during device preparation. Fortunately, controlling the thickness at the micron level in deep silicon etching of MEMS is no longer a problem.

Figure 1g shows the calculated enhancement of the electric field intensity at the terahertz detector with *d*_substrate_ = 510 μm in the DBR for three cases: (1) only the substrate layer, (2) the substrate layer with one Si/air layer DBR on top, and (3) the substrate layer with three Si/air layers DBR on top. At 0.479 THz with no DBR, |*E*_d_|^2^/|*E*_i_|^2^ is only 3.8 and the FWHM is 15,300 MHz. When the DBR has one Si/air layer, |*E*_d_|^2^/|*E*_i_|^2^ increases to 24 and the FWHM is 2250 MHz. With three Si/air layers, |*E*_d_|^2^/|*E*_i_|^2^ is 57 and the FWHM is only 295 MHz. Notably, the resonant frequencies are consistent with the resonant frequencies of the structure with only the substrate layer. That is, the thickness of the detector chip determines the resonant frequencies of the entire cavity in the band gap of DBR, and this is consistent with the previous analysis. The corresponding reflectance of these three structures is also calculated, and to further illustrate these characteristics, we also calculated the reflection of a three-layer Si/air DBR hybrid Tamm cavity for different substrate thicknesses (Supplementary Note S3). The resonant modes shift to lower frequencies as the substrate thickness increases. This is the so-called redshift, which is consistent with the case with the substrate layer only.

The electric field at the position of the detector (|*E*_d_|) is the best figure of merit. Figure 1h shows the calculated *E*_d_ in the hybrid Tamm cavity for different thicknesses of the detector substrate (*d*_substrate_) and frequencies from 0.25 to 0.60 THz. *E*_d_ increases significantly at the resonance point, and the resonance is strong in accordance with Eq. (2). Prominently, there are five cavity modes, corresponding to *N* = 2, 3, 4, 5, and 6 in Eq. (2), as indicated by the colored dashed lines. The horizontal white dashed line indicates |*E*_d_| for the substrate layer at *d*_substrate_ = 510 μm, and the white dots are |*E*_d_| at *A*, *B*, and *C*. Clearly, the resonant modes of the hybrid Tamm cavity can be adjusted by changing the substrate thickness. The resonance shifts to a higher frequency when *d*_substrate_ is decreased, which is a blueshift, and the resonance frequencies overlapped, meaning that the low resonance mode of a thin substrate overlaps with the high resonance mode of a thick substrate, as shown in Fig. 1i. The splitting observed within the 0.35–0.4 THz region has anti-crossing effect in Fig. 1h, indicating a hybridization mode which is the strong coupling of FP cavity mode excited in detector substrate and leaky Tamm mode excited in a pure Tamm structure. As analyzed in Supplementary Note S3, this splitting is caused by the coupling between the leaky Tamm cavity mode and the detector’s substrate cavity (FP mode). The leaky Tamm modes has low quality factor *Q* and large reflectivity due to the imperfect reflection outside the DBR stopband^56,73^, localizes its energy within the DBR structure, giving space for the detector substrate to excite FP cavity modes. Consequently, the hybrid mode exhibits leakiness, leading to lower electrical intensity.

When designing this kind of hybrid Tamm cavity, the target resonance points can first be calculated directly from the corresponding resonant modes of the detector chip only. Then the DBR can be designed to excite Tamm modes to couple with these FP cavity modes, enhancing the electric field at the detector without changing the resonant frequency points of the entire structure. Moreover, the resonant bandwidth can be narrowed. The detector chip and dielectric DBR are designed separately and then assembled together, which is convenient for design and fabrication. This all-silicon hybrid Tamm cavity can be used as a general platform for terahertz sources, detectors, and other functional devices. It is, possibly, the ultimate solution for achieving strong interactions between terahertz electromagnetic waves and matter.

### Device fabrication

**Fig. 2:**
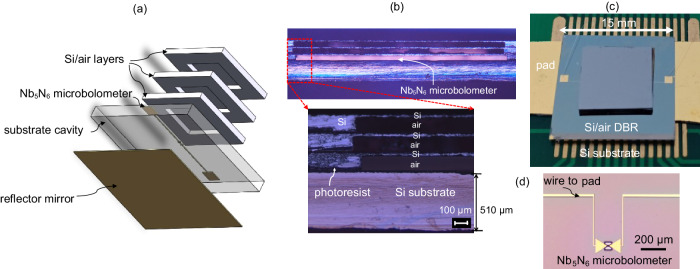
Pictures of components of the prepared Tamm-cavity terahertz detector. **a** 3D stereogram of the hybrid Tamm-cavity detector, which consists of a DBR with three Si/air layers and a Nb_5_N_6_ microbolometer detector. There is a metal reflector on the back of the detector chip. **b** Side view of Si/air DBR layers assembled onto the detector chip after being bonded together with a photoresist. **c** Package for a hybrid Tamm-cavity detector on a printed circuit board. **d** Nb_5_N_6_ microbolometer terahertz detector.

To realize the hybrid Tamm-cavity structure with a detector embedded in, a 6-inch HRFZ-Si wafer (ρ > 10,000 Ω.cm) is thinned to 148 μm. An array of air cavities with a 33-μm top layer of silicon and a 115-μm bottom layer of air is formed by deep silicon etching of the same wafer. The unit size is 9 mm × 9 mm. Considering the spot size of an incident terahertz wave, the area of the opening in a silicon pixel is 5 mm × 5 mm, as shown in Fig. 2a. We cut the wafer into many single-pixel Si/air layer blocks, which are then stacked to form a multilayer DBR using a photoresist, as shown in Fig. 2a. The thickness of the photoresist distributed around the silicon support leg is about 1 μm, which has little influence on the entire hybrid Tamm cavity. For the detector chip, we used a 510-μm HRFZ-Si substrate. The Nb_5_N_6_ microbolometer is micro-fabricated through magnetron sputtering, lithography, air-bridge etching, and other micro-processing techniques. Figure 2d is an optical photograph of the finished detector chip. The DBR chip and the detector chip are micro-assembled together by a photoresist to form the hybrid Tamm cavity. Figure 2b is a side view of the hybrid Tamm-cavity detector. To read out the response voltage of the detector, the entire package is fixed to a printed circuit board [Fig. 2c]. The preparation of the hybrid Tamm-cavity detector is illustrated in detail in Supplementary Note S4.

The multilayer DBR is obtained by stacking Si/air layer blocks, which were from the same wafer, by deep silicon etching as a MEMS process. Furthermore, the detector chips and the DBR chips are prepared separately and can be assembled or disassembled. Fabricating this kind of hybrid Tamm-cavity structure is compatible with the fabrication of other terahertz functional devices, and thus, it provides an excellent platform for enhancing the interactions between terahertz waves and matter. In particular, there are many potential applications due to the strong electromagnetic coupling between the terahertz waves and the two-dimensional material.

### THz response of the Tamm-cavity detector

**Fig. 3:**
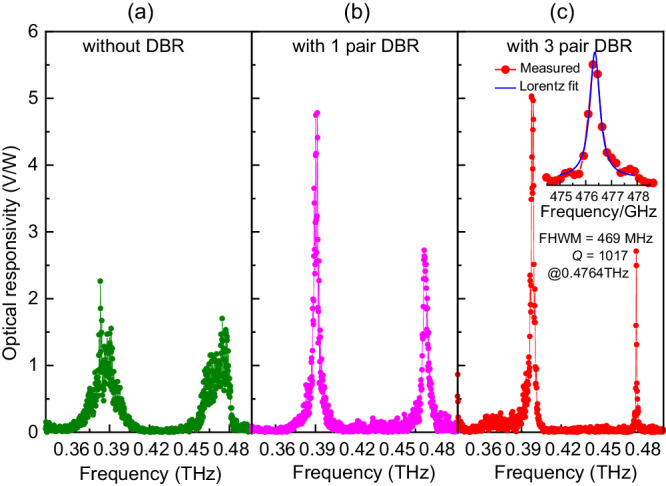
Optical voltage responsivities of detectors. **a** with a zero-layer DBR, (**b**) with a one-layer DBR, and (**c**) with a three-layer DBR. The inset is a magnified view near the resonant mode at 0.476 THz.

To verify the design of the proposed Tamm-cavity terahertz detector, three cavities coupled to a Nb_5_N_6_ microbolometer detector were prepared: (1) only the substrate layer (without a DBR), (2) a one-layer Si/air DBR, and (3) a three-layer Si/air DBR. Figure 3 shows the measured optical voltage responsivity of these three detectors. The measurement setup and method are described in Methods. The Si/air photonic crystal layers in this hybrid Tamm cavity significantly increases the interaction between an incident terahertz wave and the sensor, but hardly changes the resonant modes of the detector. These results are consistent with our previous simulation analysis. The findings are one of the subtleties of a hybrid Tamm cavity.

**Fig. 4:**
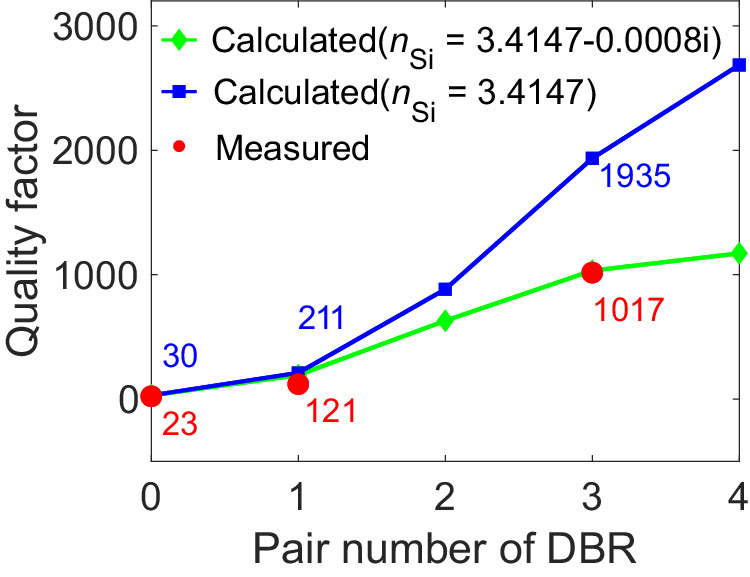
Comparison of measured and calculated *Q* values at a resonant mode (0.48 THz) of the detector with different pair numbers of DBR. The calculated *Q* values are extracted from reflection spectrum with *n*_Si_ = 3.4147 and *n*_Si_ = 3.4147–0.0008i, respectively.

Figure 3a shows the optical voltage responsivity of the detector without a DBR. As discussed above, due to the cavity modes in the substrate layer, the response has two resonant peaks at 0.40 and 0.48 THz. The FWHM at 0.48 THz is 20.6 GHz, and the *Q* value is 23, as calculated by Lorentz fitting. Figure 3b shows the optical voltage responsivity when the detector has a one-layer DBR. It also resonates at about 0.40 and 0.48 THz. There is a twofold increase in the optical voltage responsivity at 0.40 THz and a 1.5- fold increase at 0.48 THz. The FWHM is 3.90 GHz, and the *Q* value was 121 at 0.48 THz, both of which had improved by 5.3 times compared with the substrate layer only. Figure 3c shows the optical voltage responsivity when the detector has a three-layer DBR. This detector also resonates at about 0.40 and 0.48 THz. Based on Lorentz fitting, the response bandwidth and *Q* value reached 469 MHz and 1017, respectively, as shown in the inset. To the best of our knowledge, this is the narrowest bandwidth for a terahertz detector that has been reported. Note that the optical voltage responsivity of the detector is only a little higher than that of the one-layer DBR. The escalation of dielectric loss is primarily attributed to the increasing number of DBR layers. Furthermore, deviations in the thickness of each layer and surface roughness stemming from the MEMS process notably hinder the hybrid Tamm cavity’s performance. In our calculation, a mere ±3 μm fluctuation in layer thicknesses resulted in up to a 7% deviation in *Q*. Potential roughness on the silicon surface induces diffuse reflections, limiting the amount of electromagnetic wave that can be coupled to Au mirror, ultimately leading to a diminished *Q* and electrical intensity. The voltage responses are almost zero at non-resonant frequencies, which verifies the perfect filtering characteristics of the hybrid Tamm cavity. Table 1 compares the measured and calculated *Q* values and FWHM values at the resonant modes for the three detectors. The positions of the measured resonance peaks are almost the same as the calculated peaks. There was a slight deviation in frequency, mainly caused by errors when tuning the thickness of the DBR layers. The calculated results are for an ideal situation that neglects absorption by the layers. As shown in Fig. 4, the deviation of the *Q* values increases as the number of layers increases. The theoretical *Q* value reaches 1935, but the measured value is only 1017 for the detector with a three-layer DBR. Obviously, the measured results did not achieve the quality of the theoretical values, mainly because the dielectric losses are not taken into consideration in the calculations. To analyze the effects of dielectric losses, the reflectance of the hybrid Tamm cavity is calculated with dielectric constants of the HRFZ-Si with different imaginary parts (Supplementary Note 5). The calculations show that the hybrid Tamm cavity is sensitive to the refractive index, and the resonant frequency and reflectance have a strong dependence on the permittivity of the HRFZ-Si and the metal. Moreover, the terahertz source is tuned to a resolution of 0.18 GHz in the experiment and the frequency interval used in the simulation was 0.1 GHz, which may also be why the measured *Q* value is not as high as the calculated value.

**Table 1:** Comparison between measured and calculated resonant frequency and FWHM

DBR pair	Resonant frequency (THz)		FWHM (MHz)	
	Cal.	Mea.	Cal.	Mea.
0	0.473	0.474	14200	20609
1	0.478	0.472	2286	3901
3	0.479	0.476	247	469

### Tunability response of the Tamm-cavity detector

**Fig. 5:**
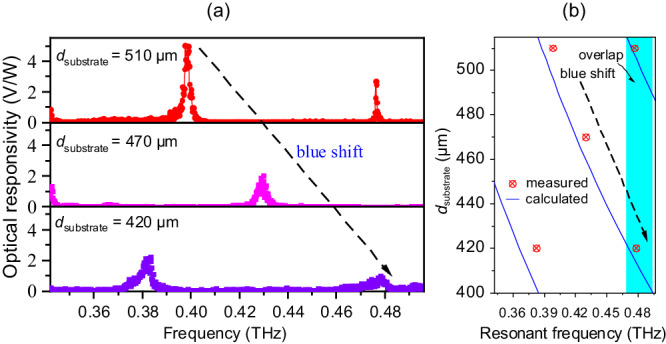
Demonstration of the tunability of the hybrid Tamm-cavity terahertz detectors. **a** Measured optical responsivity of Tamm-cavity detectors with *d*_substrate_ = 510, 470, or 420 μm. **b** Comparison of the measured and calculated resonant frequencies for *d*_substrate_ from 510 to 420 μm. The black dashed arrow indicates the blueshift, and the cyan region is where the cavity modes overlap.

To illustrate that the resonant modes of the detectors with a hybrid Tamm cavity can be tuned by controlling the substrate thickness (*d*_substrate_) of the detector chip, the substrate is mechanically thinned from 510 to 470 μm and then to 420 μm, and assembled with the same three Si/air layers. The measured optical voltage responsivities of these detectors are shown in Fig. 5a. As *d*_substrate_ decreased black dashed arrow in [Fig. 5a], the resonant frequency of the detector became higher, which is consistent with our calculated results [Fig. 1i]. Within the range of measured frequencies, the resonant frequency with a substrate thickness of 510 μm corresponds to the resonant modes *N* = 4 and 5 in the substrate layer. The resonant frequency with a substrate thickness of 470 μm corresponds to the resonant mode *N* = 4 in the substrate layer. The resonant frequency with a substrate thickness of 420 μm corresponds to the resonant modes *N* = 3 and 4. The high resonant mode in the hybrid Tamm cavity with a thick substrate overlaps with the low resonant mode with a thin substrate.

To verify the accuracy of the above design and analysis, in particular to demonstrate the tunability and overlap of the cavity modes, the measured resonant frequencies of the hybrid Tamm-cavity detector with different values of *d*_substrate_ [red circled crosses] and the calculated resonant frequencies [extracted from Fig. 1h and shown as blue lines] are plotted in Fig. 5b. The measured and calculated values match very well. The black dashed arrow indicates the blueshifts, and the cyan region is where the cavity modes overlap. Tunable detection can be realized with this hybrid Tamm cavity just by mechanically thinning the substrate and assembling the DBR. Moreover, the signals from other bands can be filtered out by the cavity detection system. Due to the non-negligible dielectric loss and absorption in the substrate layer, the *Q* value and bandwidth have both significantly deteriorated compared to the theoretical values [Fig. 1i]. Still, this is the pioneering report of a hybrid Tamm cavity terahertz detector, and it achieves an ultra-high resonant *Q* value and narrow response bandwidth experimentally.

## Discussion

We demonstrated a terahertz detector integrated into a Tamm cavity. The detector chip, positioned between a multilayer Si/air DBR and an Au reflector, exhibits significantly enhanced interaction with terahertz signals within this hybrid Tamm cavity. At the resonance wavelength of the Tamm mode, the Au film and top DBR trap light effectively in the cavity, resulting in local enhancement of electric field at the detector. The detector achieves an exceptional *Q* (*Q* = 1017) with an extraordinarily narrow bandwidth (FWHM = 469 MHz). The *Q* of this hybrid structure surpasses that of a pure Tamm cavity and a Fabry-Perot cavity. The ability to fine-tune the frequency for a narrow bandwidth by adjusting *d*_substrate_ makes this approach highly promising for developing terahertz spectrometers. The presented hybrid Tamm cavity, achievable through straightforward MEMS processing, stacking, and assembly without altering the device’s original resonant frequency, offers a simplified design and implementation. The versatility of this hybrid Tamm-cavity terahertz detector extends to enhancing the performance of various terahertz devices, particularly in the realm of high-power sources, high-sensitivity detectors, and high-performance functional devices. Furthermore, its application holds promise for groundbreaking investigations into the strong coupling between 2D materials and terahertz waves.

## Methods

### Experimental setup and optical responsivity characterization

For the optical responsivity measurements, the detector under test was biased by a dc current (0.4 mA). The radiation was focused by two off-axis parabolic mirrors to yield the largest possible signal from the detector. For the alignment, a laser beam was used for rough adjustment, and then the detector was moved until its response voltage was a maximum. The photovoltage data were collected by a lock-in amplifier (SR830). The terahertz radiation source from 0.34 to 0.50 THz was obtained using multipliers in series (Agilent E8257D microwave source + VDI-AMC-336 + WR4.3 × 2 + WR2.2 × 2). The output power of the terahertz source was about 50 μW, which was varied with the signal frequency. It was modulated using a 4-kHz TTL signal. A thermal sensor (3A-P-THz, Ophir) was used to calibrate the optical responsivity as *R*_O_ = *V* / *P*, where *P* is the total incident power and *V* is the output voltage of the detector. To make it easier to compare and explain the responses of detectors with different cavity structures, the entire power incident on the detector was simply assumed to be effectively absorbed by the microbolometer. All measurements were performed in air at room temperature^81,82^.

### Numerical simulations

TMM and electromagnetic simulation software (FDTD) are applied to calculate the reflectivity spectra associated with the profiles of the intensity enhancement of the electric field. In the simulations, the permittivity of metal Au is described using the Drude model:

$$\varepsilon (\omega )={\varepsilon }_{\infty }+\frac{{\omega }_{p}^{2}}{i\omega \gamma -{\omega }^{2}}$$

where $${\varepsilon }_{\infty }=4.8952$$, $${\omega }_{P}/2\pi=2126.4\,{{{{{\rm{THz}}}}}}$$, $$\gamma /2\pi=19.6\,{{{{{\rm{THz}}}}}}$$, and $${n}_{M}=\sqrt{\varepsilon (\omega )}$$.

In the simulation, the refractive indices of the other materials (e.g., HRFZ-Si) were also from ref. ^79^.

### Supplementary information

Supplementary Information

Peer Review File

## Data Availability

The authors declare that all relevant data are available in the paper and Supplementary Information, or from the corresponding author on request.

## References

[1] Ko¨hler R (2002). Terahertz semiconductor-heterostructure laser. Nature.

[2] Mahler L (2009). Vertically emitting microdisk lasers. Nat. Photon.

[3] Biasco S (2018). Continuous-wave highly-efficient low-divergence terahertz wire lasers. Nat. Commun..

[4] Chevalier P (2019). Widely tunable compact terahertz gas lasers. Science.

[5] Vicarelli L (2012). “Graphene fieldeffect transistors as room-temperature terahertz detectors,”. Nat. Mater..

[6] Palaferri D (2015). Patch antenna terahertz photodetectors. Appl. Phys. Lett..

[7] Okamoto K (2017). Terahertz sensor using photonic crystal cavity and resonant tunneling diodes. J. Infrared Millimeter Terahertz Waves.

[8] Paulillo B (2017). Ultrafast terahertz detectors based on three-dimensional meta-atoms. Optica.

[9] Bandurin DA (2018). Resonant terahertz detection using graphene plasmons. Nat. Commun..

[10] Gayduchenko I (2021). Tunnel field-effect transistors for sensitive terahertz detection. Nat. Commun..

[11] Chen HT (2009). A metamaterial solid-state terahertz phase modulator. Nat. Photon.

[12] Meijer A (2016). An ultrawide-bandwidth single-sideband modulator for terahertz frequencies. Nat. Photon.

[13] Ummethala S (2019). THz-to-optical conversion in wireless communications using an ultra-broadband plasmonic modulator. Nat. Photonics.

[14] Sengupta K, Nagatsuma T, Mittleman DM (2018). Terahertz integrated electronic and hybrid electronic–photonic systems. Nat. Electron..

[15] Headland D, Yu X, Fujita M, Nagatsuma T (2019). Near-field out-of-plane coupling between terahertz photonic crystal waveguides. Optica.

[16] Mittleman DM (2018). Twenty years of terahertz imaging [Invited]. Opt. Express.

[17] Withayachumnankul W (2007). T-ray sensing and imaging. Proc. IEEE.

[18] Stantchev RI (2020). Real-time terahertz imaging with a single-pixel detector. Nat. Commun..

[19] Jepsen PU, Cooke DG, Koch M (2011). Terahertz spectroscopy and imaging—modern techniques and applications. Laser Photonics Rev..

[20] Du S (2018). Terahertz dynamics of electron–vibron coupling in single molecules with tunable electrostatic potential. Nat. Photon.

[21] Hindle F, Bocquet R, Pienkina A, Cuisset A, Mouret G (2019). Terahertz gas phase spectroscopy using a high-finesse Fabry-Pérot cavity. Optica.

[22] Koenig S (2013). Wireless sub-THz communication system with high data rate. Nat. Photon.

[23] Nagatsuma T, Ducournau G, Renaud CC (2016). Advances in terahertz communications accelerated by photonics. Nat. Photon..

[24] Ma J (2018). Security and eavesdropping in terahertz wireless links. Nature.

[25] Yang Y (2020). Terahertz topological photonics for on-chip communication. Nat. Photonics.

[26] Mittleman DM (2017). Perspective: terahertz science and technology. J. Appl. Phys..

[27] Todorov Y (2009). Strong light–matter coupling in subwavelength metal-dielectric microcavities at terahertz frequencies. Phys. Rev. Lett..

[28] Dyer GC (2012). Inducing an incipient terahertz finite plasmonic crystal in coupled two dimensional plasmonic cavities. Phys. Rev. Lett..

[29] Davoyan AR, Popov VV, Nikitov SA (2012). Tailoring terahertz near-field enhancement via two-dimensional plasmons. Phys. Rev. Lett..

[30] Scalari G (2012). Ultrastrong coupling of the cyclotron transition of a 2D electron gas to a THz metamaterial. Science.

[31] Kampfrath T, Tanaka K, Nelson K (2013). Resonant and nonresonant control over matter and light by intense terahertz transients. Nat. Photon.

[32] Kakimi R (2014). Capture of a terahertz wave in a photonic-crystal slab. Nat. Photon.

[33] Zhang Q (2016). Collective non-perturbative coupling of 2D electrons with high-quality-factor terahertz cavity photons. Nat. Phys..

[34] Feres FH (2021). Sub-diffractional cavity modes of terahertz hyperbolic phonon polaritons in tin oxide. Nat. Commun..

[35] Bahsoun H, Chervy T (2018). Electronic light–matter strong coupling in nanofluidic fabry–pérot cavities. ACS Photonics.

[36] Ünlü MSelim, Strite S (1995). Resonant cavity enhanced photonic devices. J. Appl. Phys..

[37] Deng H, Weihs G, Santori C, Bloch J, Yamamoto Y (2002). Condensation of semiconductor microcavity exciton polaritons. Science.

[38] Brückner R (2012). Phase-locked coherent modes in a patterned metal–organic microcavity. Nat. Photon.

[39] Kavokin, A., Baumberg, J., Malpuech, G. & Laussy, F. Microcavities (Oxford University Press, Oxford, 2011).

[40] Wang Z, Zhang B, Deng H (2015). Dispersion engineering for vertical microcavities using subwavelength gratings. Phys. Rev. Lett..

[41] Yoshie T (2004). Vacuum Rabi splitting with a single quantum dot in a photonic crystal nanocavity. Nature.

[42] Baba T (2008). Slow light in photonic crystals. Nat. Photon.

[43] Mühlschlegel P, Eisler H-J, Martin OJF, Hecht B, Pohl DW (2005). Resonant optical antennas. Science.

[44] Novotny L, van Hulst N (2011). Antennas for light. Nat. Photon.

[45] Forn-Díaz P, Lamata L, Rico E, Kono J, Solano E (2019). Ultrastrong coupling regimes of light–matter interaction. Rev. Mod. Phys..

[46] Ohno H (1990). Observation of ‘Tamm states’ in superlattices. Phys. Rev. Lett..

[47] Kaliteevski M (2007). Tamm plasmon-polaritons: possible electromagnetic states at the interface of a metal and a dielectric Bragg mirror. Phys. Rev. B.

[48] Sasin M (2008). Tamm plasmon polaritons: slow and spatially compact light. Appl. Phys. Lett..

[49] Brückner R (2011). Hybrid optical Tamm states in a planar dielectric microcavity. Phys. Rev. B.

[50] Afinogenov BI, Bessonov VO, Nikulin AA, Fedyanin AA (2013). Observation of hybrid state of Tamm and surface plasmon-polaritons in one-dimensional photonic crystals. Appl. Phys. Lett..

[51] Furchi M (2012). Microcavity-integrated graphene photodetector. Nano Lett..

[52] Sobhani A (2013). Narrowband photodetection in the near-infrared with a plasmon-induced hot electron device. Nat. Commun..

[53] Mischok A (2017). Controlling Tamm plasmons for organic narrowband near-infrared photodetectors. ACS Photonics.

[54] Eizner E, Brodeur J, Barachati F, Sridharan A, Kéna-Cohen S (2018). Organic photodiodes with an extended responsivity using ultrastrong light–matter coupling. ACS Photonics.

[55] Chen Yikai (2014). Tamm plasmon- and surface plasmon-coupled emission from hybrid plasmonic–photonic structures. Optica.

[56] Wang Zhiyu, K. Clark J (2018). Narrowband thermal emission realized through the coupling of cavity and tamm plasmon resonances. ACS Photonics.

[57] Toanen V (2020). Room-temperature lasing in a low-loss tamm plasmon cavity. ACS Photonics.

[58] Grossmann C (2011). Tuneable polaritonics at room temperature with strongly coupled Tamm plasmon, polaritons in metal/air-gap microcavities. Appl. Phys. Lett..

[59] Gubaydullin AR (2017). Tamm plasmon sub-wavelength structuration for loss reduction and resonance tuning. Appl. Phys. Lett..

[60] Ferrier L (2019). Tamm plasmon photonic crystals: from bandgap engineering to defect cavity. APL Photonics.

[61] Coquillat D (2016). Improvement of terahertz field effect transistor detectors by substrate thinning and radiation losses reduction. Opt. Express.

[62] Hou HW, Liu Z, Teng JH, Palacios T, Chua SJ (2017). Enhancement of responsivity for a transistor terahertz detector by a Fabry-Pérot resonance-cavity. Appl. Phys. Lett..

[63] Duan G (2018). Analysis of the thickness dependence of metamaterial absorbers at terahertz frequencies. Opt. Express.

[64] Qin Q (2009). Tuning a terahertz wire laser. Nat. Photon.

[65] Demir K, Unlu M (2020). Miniature MEMS: Novel key components toward terahertz reconfigurability. J. Microelectromech. Syst..

[66] Castellano F (2015). Tuning a microcavity-coupled terahertz laser. Appl. Phys. Lett..

[67] Curwen CA, Reno JL, Williams BS (2019). Broadband continuous single-mode tuning of a short-cavity quantum-cascade VECSEL. Nat. Photonics.

[68] Dong HY, Wang J, Cui TJ (2013). One-way Tamm plasmon polaritons at the interface between magnetophotonic crystals and conducting metal oxides. Phys. Rev. B.

[69] Hu Jigang (2019). Strong longitudinal coupling of Tamm plasmon polaritons in graphene/DBR/Ag hybrid structure. Opt. Express.

[70] Sharma R (2007). Gallium-nitride-based microcavity light emitting diodes with air-gap distributed Bragg reflectors. Appl. Phys. Lett..

[71] Matioli Elison (2010). High extraction efficiency light-emitting diodes based on embedded air-gap photonic-crystals. Appl. Phys. Lett..

[72] Tao Renchun, Kamide Kenji, Arita Munetaka, Kako Satoshi, Arakawa Yasuhiko (2016). Room-temperature observation of trapped exciton-polariton emission in GaN/AlGaN microcavities with air-gap/III-Nitride distributed bragg reflectors. ACS Photonics.

[73] Messelot S (2020). Tamm cavity in the THz spectral range,. ACS Photonics.

[74] Dufferwiel S (2015). Exciton–polaritons in van der Waals heterostructures embedded in tunable microcavities. Nat. Commun..

[75] Lundt N (2016). Room-temperature Tamm-plasmon exciton-polaritons with a WSe_2_ monolayer. Nat. Commun..

[76] Schneider C (2018). Two-dimensional semiconductors in the regime of strong light-matter coupling. Nat. Commun..

[77] Horng J (2019). Engineering radiative coupling of excitons in 2D semiconductors. Optica.

[78] Kawano Y, Ishibashi K (2008). An on-chip near-field terahertz probe and detector. Nat. Photon.

[79] Dai J, Zhang J, Zhang W, Grischkowsky D (2004). Terahertz time-domain spectroscopy characterization of the far-infrared absorption and index of refraction of high-resistivity, float-zone silicon. J. Opt. Soc. Am. B.

[80] Tsuruda K, Fujita M, Nagatsuma T (2015). Extremely low-loss terahertz waveguide based on silicon photonic-crystal slab. Opt. Express.

[81] Tu X (2019). Fabry–Pérot cavity-coupled microbolometer terahertz detector with a continuously tunable air spacer gap. Opt. Lett..

[82] Tu X (2018). Investigation of antenna-coupled Nb_5_N_6_ microbolometer THz detector with substrate resonant cavity. Opt. Express.

