# Supplementary material for: Tamm-cavity terahertz detector
Source: Nat Commun. 2024 Jul 2;15:5542. doi: 10.1038/s41467-024-49759-z (PMC11219876; doi:10.1038/s41467-024-49759-z)
Supplement: Supplementary file 1 — Supplementary Information [file 41467_2024_49759_MOESM1_ESM.pdf]

Supplementary material for

Tamm-cavity terahertz detector

Xuecou Tu[1,2,*], Yichen Zhang[1], Shuyu Zhou[1], Wenjing Tang[1], Xu Yan[1], Yunjie Rui[1], Wohu Wang[1], Bingnan Yan[1], Chen Zhang[1], Ziyao Ye[1], Hongkai Shi[1], Runfeng Su[1], Chao Wan[3], Daxing Dong[4], Ruiying Xu[5], Qing-Yuan Zhao[1,3], La-Bao Zhang[1,2], Xiao-Qing Jia[1,2], Huabing Wang[1,3], Lin Kang[1,2,*], Jian Chen[1,3] and Peiheng Wu[1,2,*]

[1]*Research Institute of Superconductor Electronics (RISE), School of Electronic Science and Engineering, Nanjing University, Nanjing, Jiangsu 210023, China*

[2]*Hefei National Laboratory, Hefei 230088, China*

[3]*Purple Mountain Laboratories, Nanjing, Jiangsu 211111, China*

[4]*Department of Applied Physics, Nanjing University of Aeronautics and Astronautics, Nanjing 210016, China*

[5]*Nanjing Electronic Devices Institute, Nanjing, 210016, China*

Correspondence should be addressed to Xuecou Tu (tuxuecou@nju.edu.cn) and Lin Kang (kanglin@nju.edu.cn) and Peiheng Wu (phwu@nju.edu.cn)

The PDF file includes:

Supplementary Note S1 to S5

Supplementary Note S1: Reflection spectra calculated by the transfer-matrix method

Transfer-matrix method

The transfer-matrix method can be used to obtain parameters such as the reflectance and transmittance of incident light in a multilayer planar dielectric structure. A metal-DBR cavity composed of silicon and air layers is shown in Fig. S1.

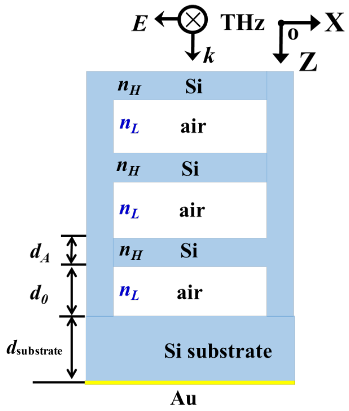

Fig. S1. Electromagnetic model of the metal-DBR cavity.

For simplicity, only the case of normal incidence of the electromagnetic wave is considered. A monochromatic plane incident wave can be described by:

$$E_i = E_0 \exp(-ik_0 z) \tag{1}$$

where E_0 is the amplitude of the incident electric field and k_0 is the wave number of the incident light in vacuum. The reflected light can be described by:

$$E_r = rE_0 \exp(ik_0 z) \tag{2}$$

where r is the amplitude reflection coefficient of the beam at the interface.

The electric field in any dielectric layer can be viewed as the superposition of the incident and reflected electric fields.

$$E_m^+ = C_m E_0 \exp(-ikz) \tag{3}$$

$$E_m^- = D_m E_0 \exp(ikz) \tag{4}$$

According to the Maxwell equations and boundary conditions, we can obtain the form of the transfer matrix at each interface. At the interface between air and the first layer of silicon ($z = 0$):

$$1 + r = C_1 + D_1 \tag{5}$$

$$\frac{k_0}{\mu_0}(1-r) = \frac{k_A}{\mu_A}(C_1 - D_1) \tag{6}$$

where μ_0 is the permeability of a vacuum and μ_A is the relative permeability of silicon. μ_A is generally equal to 1. A matrix relationship can be established at the $z = 0$ interface:

$$\begin{bmatrix} 1 \\ r \end{bmatrix} = M_{OA1} \begin{bmatrix} C_1 \\ D_1 \end{bmatrix} \tag{7}$$

$$M_{OA1} = \begin{bmatrix} 1 & 1 \\ 1 & -1 \end{bmatrix}^{-1} \begin{bmatrix} 1 & 1 \\ \dfrac{k_A}{k_0 \mu_A} & -\dfrac{k_A}{k_0 \mu_A} \end{bmatrix} \tag{8}$$

According to the relationship between wave number, magnetic permeability, and refractive index, the above formula can be simplified:

$$M_{OA1} = \begin{bmatrix} 1 & 1 \\ 1 & -1 \end{bmatrix}^{-1} \begin{bmatrix} 1 & 1 \\ n_a & -n_a \end{bmatrix} \tag{9}$$

where n_A is the refractive index of silicon. The boundary conditions at the $z = d_A$ interface:

$$\begin{bmatrix} C_1 \\ D_1 \end{bmatrix} = M_{AO} \begin{bmatrix} C_2 \\ D_2 \end{bmatrix} \tag{10}$$

$$M_{AO} = \begin{bmatrix} \exp(-ik_A d_A) & 0 \\ 0 & \exp(ik_A d_A) \end{bmatrix}^{-1} \begin{bmatrix} 1 & 1 \\ 1 & -1 \end{bmatrix}^{-1} \begin{bmatrix} 1 & 1 \\ 1/n_A & -1/n_A \end{bmatrix} \tag{11}$$

where d_A is the thickness of the silicon layer. Similarly, the matrix relation when the electromagnetic wave propagates from the air layer to the silicon layer can be obtained:

$$M_{OA} = \begin{bmatrix} \exp(-ik_o d_o) & 0 \\ 0 & \exp(ik_o d_o) \end{bmatrix}^{-1} \begin{bmatrix} 1 & 1 \\ 1 & -1 \end{bmatrix}^{-1} \begin{bmatrix} 1 & 1 \\ n_A & -n_A \end{bmatrix} \tag{12}$$

where d_O is the thickness of the air layer.

At the interface between the defect layer and gold:

$$M_{AD} = \begin{bmatrix} \exp(-ik_A d_{\text{substrate}}) & 0 \\ 0 & \exp(ik_A d_{\text{substrate}}) \end{bmatrix}^{-1} \begin{bmatrix} 1 & 1 \\ 1 & -1 \end{bmatrix}^{-1} \begin{bmatrix} 1 & 1 \\ n_m / n_A & -n_m / n_A \end{bmatrix} \tag{13}$$

where $d_{\text{substrate}}$ is the thickness of the substrate layer and n_m is the refractive index of gold. The entire transfer matrix can be written as:

$$M = M_{OA1} M_{AO} M_{OA} M_{AO} M_{OA} M_{AO} M_{OA} M_{AD} \tag{14}$$

Furthermore, it can be expressed as:

$$M = \begin{bmatrix} M_{11} & M_{12} \\ M_{21} & M_{22} \end{bmatrix} \tag{15}$$

The amplitudes of the reflection coefficient (r) and transmission coefficient (t) are expressed as:

$$r = \frac{M_{21}}{M_{11}}, \quad t = \frac{1}{M_{11}} \tag{16}$$

Once the reflectivity of a monochromatic electromagnetic wave is found, the reflection spectrum in a certain band can be obtained with the same method.

Calculation of the reflection spectrum of the metal-DBR cavity by TMM

The hybrid Tamm cavity presented in this paper is prepared by alternately stacking silicon layers and air layers on a detector chip with a gold reflective mirror. The substrate of the detector chip (dielectric layer next to the gold) is silicon, which has a high refractive index. The calculation results of the reflective spectrum of the hybrid Tamm cavity mentioned above with different substrate cavity thickness by TMM are as follows:

(1) When the thickness of the substrate layer is the same as that of the silicon layer in the DBR, namely, $d_{substrate}$ = 33.45 μm, the dip of the reflected spectrum is around 0.65 THz, which is equal to the center frequency set by the DBR only (Fig. S2).

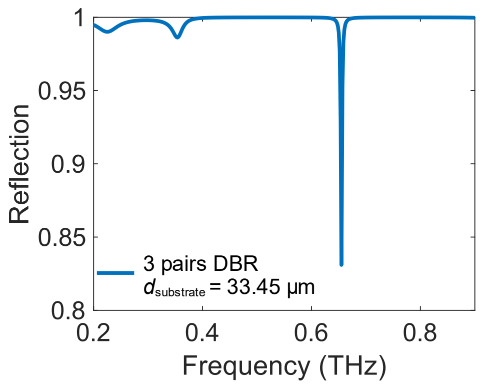

Fig. S2. Reflection spectrum of the cavity at $d_{substrate}$ = 33.45 μm. The thickness of the substrate layer is the same as that of the silicon layer in the DBR.

(2) When $d_{substrate}$ is decreased, the dip of the reflection spectrum will be blue shifted (Fig. S3).

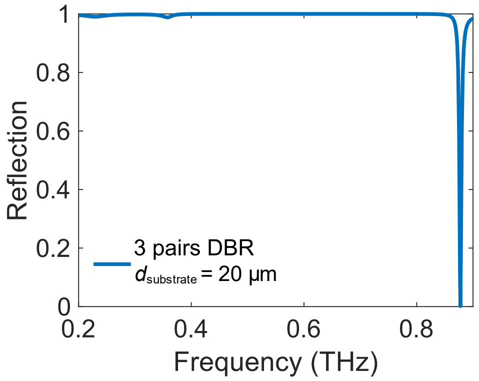

Fig. S3. Reflection spectrum of the substrate at $d_{substrate}$ = 20 μm.

(3) In contrast, when $d_{substrate}$ is increased, the dip of the reflection spectrum is redshifted (Fig. S4).

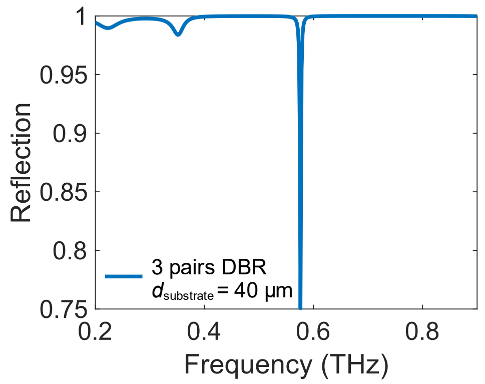

Fig. S4. Reflection spectrum of the substrate at $d_{substrate}$ = 40 µm.

(4) When d_{cavity} is increased further, multiple dips appear simultaneously in the reflection spectrum. The thickness of substrate is thick enough to excite cavity modes outside the DBR stopband, which are coupling to the leaky Tamm modes to form hybrid modes. Therefore, the number of the hybrid modes and the position of the dips depend on the thickness of the substrate ($d_{substrate}$) (Fig. S5).

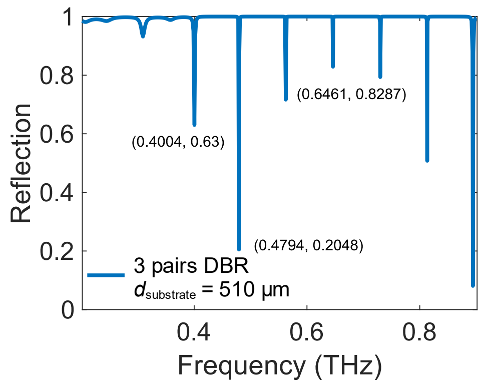

Fig. S5. Reflection spectrum of the substrate at $d_{substrate}$ = 510 µm. Three dip positions of great concern are marked.

Through the above calculations, it can be seen that the number of Tamm-cavity modes and the position of the resonant points strongly depend on the thickness of the substrate layer ($d_{substrate}$).

Supplementary Note S2: Proof of the conditions for the existence of Tamm states in the proposed hybrid Tamm cavity

As shown in Fig. S6, in the hybrid Tamm cavity, the substrate layer is modeled as a generalized microcavity ($d_{substrate}$). The metal on the left is equivalent to a gold mirror, and the DBR with the substrate on the right is regarded as a dielectric mirror. For simplicity, only normal incidence was considered. The characteristics of the metal-

DBR hybrid cavity that excite optical Tamm states were derived from the perspective of the phase changes.

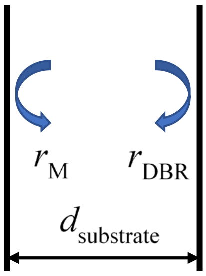

Fig. S6. Schematic of reflections at the metal interface and the DBR interface.

The total phase change of the terahertz wave in the cavity has three parts: (1) at the interface between the metal and the substrate layer, (2) between the substrate layer and the DBR interface, and (3) the phase change when the terahertz wave propagates in the substrate layer. Φ_{M} represents the phase of r_{M}, Φ_{DBR} represents the phase of r_{DBR}, and the total phase is represented by Φ:

$$\Phi = \Phi_{\mathrm{M}} + \Phi_{\mathrm{s}} + \Phi_{\mathrm{DBR}}$$

The electromagnetic wave propagates vertically in the dielectric layer, and the phase change of the round trip is:

$$\Phi_{\mathrm{s}} = \frac{4\pi n_{\mathrm{H}} d_{\mathrm{substrate}}}{\lambda}$$

The reflection coefficient r_M at the metal interface can be obtained from the Fresnel formula:

$$r_{\mathrm{M}} = \frac{n_{\mathrm{H}} - n_{\mathrm{M}}}{n_{\mathrm{H}} + n_{\mathrm{M}}}$$

where n_{H} is the refractive index of the substrate layer medium, which is HRFZ-Si so that $n_{\mathrm{H}} = 3.4147$. n_M is the refractive index of the metal. The dielectric constant of the metal Au is described by the Drude model:

$$\varepsilon(\omega) = \varepsilon_\infty + \frac{\omega_p^2}{i\omega\gamma - \omega^2}$$

where $\varepsilon_\infty = 4.8952$, $\omega_P/2\pi = 2126.4$ THz, $\gamma/2\pi = 19.6$ THz, and $n_{\mathrm{M}} = \sqrt{\varepsilon(\omega)}$.

The conditions for the existence of an optical Tamm state in the metal-DBR hybrid cavity must meet the following formula [46, 49]:

$$\Phi = \Phi_{\mathrm{M}} + \Phi_{\mathrm{s}} + \Phi_{\mathrm{DBR}} = 2N\pi$$

where *N* is a natural number.

The following are the calculated total phase for the presented hybrid Tamm cavity at 0.40, 0.48, and 0.65 THz:

(1) When $d_{\mathrm{substrate}}$ = 510 μm, it can be seen from the reflection spectrum that the frequency corresponding to the optical Tamm state is 0.40 THz. From the Drude model, $n_M = 538 - 549i$. Furthermore, the reflection coefficient $r_M = -0.9938 + 0.0063i$, $|r_{\mathrm{M}}| = 0.9938$, and the phase $\Phi_{\mathrm{M}} = 3.1352$. The reflection coefficient of the DBR, $r_{\mathrm{DBR}} = 0.4773 + 0.7957i$, can be obtained from the TMM. Also, $|r_{\mathrm{DBR}}| = 0.9279$, and the phase $\Phi_{\mathrm{DBR}} = 1.03$. The phase change of the substrate layer $\Phi_{\mathrm{s}} = 28.49$. Finally, the total phase change $\Phi = \Phi_{\mathrm{M}} + \Phi_{\mathrm{s}} + \Phi_{\mathrm{DBR}} = 32.65 \approx 10\pi$.

(2) When $d_{\mathrm{substrate}}$ = 510 μm, the frequency corresponding to the optical Tamm state is 0.48 THz. Also, $n_M = 489 - 501i$, the reflection coefficient $r_M = -0.9932 + 0.0069i$, $|r_M| = 0.9932$, the phase $\Phi_M = 3.1346$, the reflection coefficient $r_{\mathrm{DBR}} = 0.9023 + 0.4248i$, $|r_{\mathrm{DBR}}| = 0.9972$, the phase $\Phi_{\mathrm{DBR}} = 0.44$, and the phase change of the substrate layer $\Phi_{\mathrm{s}} = 34.34$. The total phase change $\Phi = \Phi_{\mathrm{M}} + \Phi_{\mathrm{s}} + \Phi_{\mathrm{DBR}} = 37.91 \approx 12\pi$.

(3) When $d_{\mathrm{substrate}}$ = 510 μm, the frequency corresponding to the optical Tamm state is 0.65 THz. Similarly, $n_M = 414 - 428i$, the reflection coefficient $r_M = -0.9920 + 0.0082i$, $|r_M| = 0.9920$, the phase $\Phi_M = 3.1333$, the reflection coefficient $r_{\mathrm{DBR}} = 0.9996$, $|r_{\mathrm{DBR}}| = 0.9996$, $\Phi_{\mathrm{DBR}} = 0$, and the phase change of the substrate layer$\Phi_s = 47.49$. The total phase change $\Phi = \Phi_{\mathrm{M}} + \Phi_{\mathrm{s}} + \Phi_{\mathrm{DBR}} = 50.623 \approx 16\pi$.

Overall, these calculations indicate that there is also an optical band-like optical Tamm state cavity in the terahertz band.

Supplementary Note S3: The analysis and discussion on the Tamm modes and it coupling with the detector's substrate cavity (FP mode)

In order to further clarify and understand the characteristics of these resonant Tamm modes, the reflection map and spatial electromagnetic field distribution of this hybrid Tamm cavity ($d_{substrate}$ = 510 μm, see Fig. S7) is calculated. Fig. S7(b) show the reflection spectrum of the hybrid Tamm cavity when $d_{substrate}$ = 510 μm, 450 μm and 33

μm respectively. The leaky Tamm modes has low quality factor Q and large reflectivity due to the imperfect reflection outside the DBR stopband [56, 73]. As shown in the inset of Fig. S7(b), within the DBR bandgap, the electric field of the hybrid cavity (at 0.47 THz) is much larger than that of the pure Tamm cavity (at 0.65 THz), almost six times larger, which confirms the highly localized enhancement and high Q value of the electric field in the hybrid Tamm cavity. Meanwhile, the existence of multiple Tamm states within the DBR bandgap is demonstrated in hybrid Tamm cavity for $d_{substrate}$ = 510 μm, 450 μm. However, in the pure Tamm cavity as demonstrated in Fig. R2, there is only one Tamm sate within the DBR bandgap and multiple leaky Tamm modes exist outside the DBR bandgap. It is found that a Tamm states is periodic resonance with the variation of the top-layer thickness of the silicon dielectric for a given wavelength for both pure and hybrid cavity structure, which is precisely determined by Formula 2 in the main text of the manuscript. As shown in Fig. S7(c), multiple splits occur at certain frequencies. This phenomenon is caused by the coupling of various leaky Tamm modes outside of the DBR stopband (Fig. S7(e), red dashed box in Fig. S7(c) and substrate cavity modes (FP modes) (Fig. S7(f)). The split and hybridization of these two modes in 0.2-1.2 THz are shown in Fig. S7(c). One of these at around 0.35 THz is enlarged and depicted in Fig. S7(d) and anti-crossing effect can be clearly seen, indicating a strong coupling. To explain the reason for the hybrid mode generated outside the band gap of DBR, we split the hybrid Tamm cavity into two parts, one of which is the pure Tamm cavity ($d_1 = \frac{\lambda}{4n_{\mathrm{Si}}}$ = 33 μm), the other part is the silicon FP cavity ($d_2 = d_{\mathrm{substrate}} - \frac{\lambda}{4n_{\mathrm{Si}}}$), and the reflection spectra of these three cavities were calculated separately. As can be seen from Fig. S7 (g), it is precisely due to the mode coupling of these two cavities that splitting and anti-crossing effect occurs.

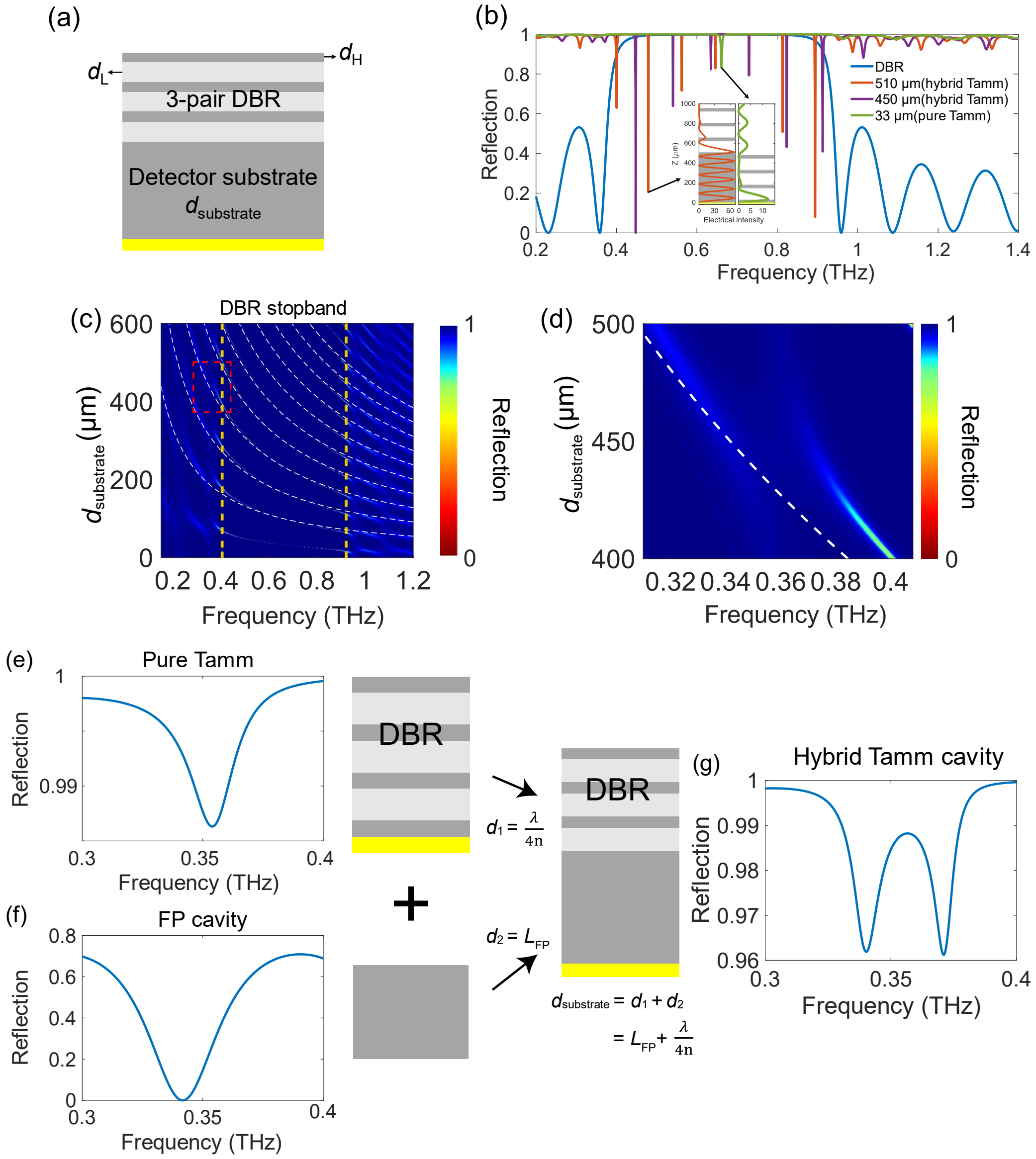

Fig. S7. The analysis and discussion on the Tamm modes and it coupling with the detector's substrate cavity. (a) The scheme of the hybrid Tamm cavity with a thickness tunable detector substrate embedded in a Tamm structure. (b) The reflection spectrum of the hybrid structure when $d_{\text{substrate}}$ = 510 μm, 450 μm and 33 μm. The inset is the calculated spatial electromagnetic field distribution of this hybrid Tamm structure ($d_{\text{substrate}}$ = 510 μm) and pure Tamm cavity ($d_{\text{substrate}} = \frac{\lambda}{4n_{Si}}$ = 33 μm). (c) The relation between the reflection and the thickness of detector substrate at 0.15 THz - 1.2 THz. Multiple anti-crossing splits occur outside the DBR stopband. (d) Enlarged image of an anti-crossing split in the red box in (c). (e) Calculated reflection of leaky Tamm mode in a pure Tamm cavity. (f) Calculated reflection of FP cavity mode in FP cavity. Considering the detector substrate is the combination of a typical DBR layer and FP cavity. The thickness of the FP cavity is $d_{\text{substrate}} - \lambda/4n_{\text{Si}}$. (g) Calculated reflection of a

coupled leaky Tamm and FP cavity mode with anti-crossing effect in a hybrid Tamm cavity.

Supplementary Note S4: Fabricating the hybrid Tamm-cavity detector

The silicon/air layers, microbolometer detector with a reflective mirror in the hybrid Tamm cavity were made separately. The air and silicon dielectric layers are made by deep silicon etching in the same high-resistivity float-zone (HRFZ) Si wafer with 4 inches, The wafer with openings array was shown in the inset at the top-right of FIG. S8. The air openings under the silicon wafer made by etching were 4 mm by 4 mm, and then the wafer was cut into blocks with size of 8 mm × 8 mm × 510 μm ($L \times W \times T$). To make the DBR, the blocks with silicon/air layers were stacked and bonded together with a photoresist to form a DBR with multiple Si/air layers. The bolometer detector chip containing a substrate layer (in the right bottom-right of FIG. S8) was made step by step, and the fabrication process in details was reported by our group before in Ref. [81, 82]. At last, the DBR block was bonded to the detector chip to form a Tamm cavity coupled detector.

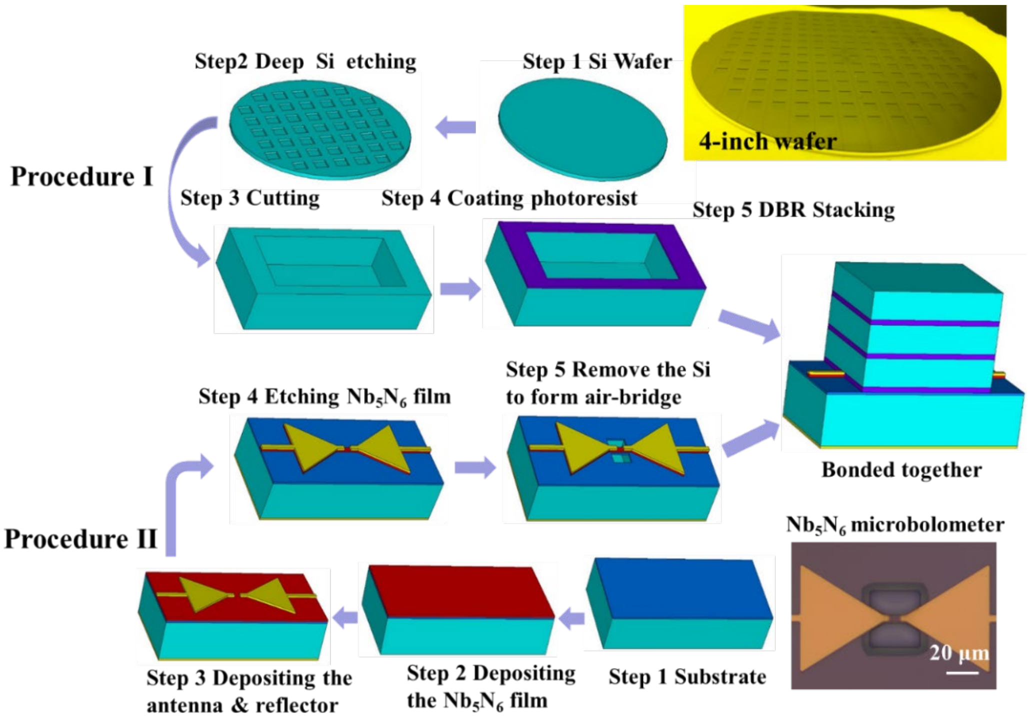

Fig. S8. Fabricating the hybrid Tamm-cavity detector.

Supplementary Note S5: Analyzing the effects of the refractive index of the HRFZ-

Si on the reflection spectrum of the hybrid Tamm cavity

Influence of the real part of the refractive index of the HRFZ-Si layer on the reflection spectrum

The real part of the refractive index of the HRFZ-Si was initially determined to be 3.4147. The effect of different values of the real part of the refractive index on the reflection spectrum was investigated. The reflection spectra calculated by TMM at $d_{\mathrm{substrate}}$ = 510 μm are plotted in Fig. S9. It can be seen that the magnitude and resonant position change slightly. There is a greater influence on the amplitude of the reflection coefficient than on the resonant frequency.

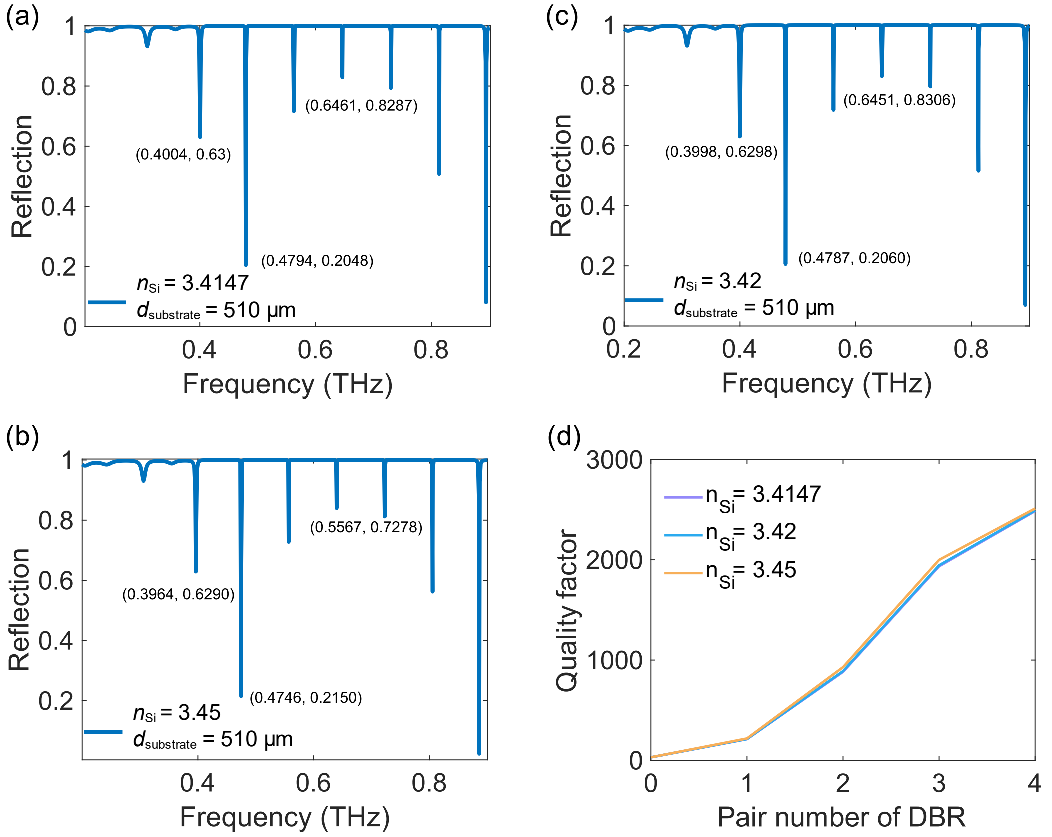

Fig. S9. Calculated reflection spectra for different real parts of the refractive index of HRFZ-Si layer at $d_{\mathrm{substrate}}$ = 510 μm: (a) n_{Si} = 3.4147, (b) n_{Si} = 3.42, and (c) n_{Si} = 3.45. (d) Calculated Q values at a resonant mode (around 0.48 THz) of the detector with different numbers of layers with n_{Si} = 3.4147, n_{Si} = 3.42 and n_{Si} = 3.45

Influence of the imaginary part of the refractive index of the HRFZ-Si layer on the reflection spectrum

In actuality, there is dielectric absorption loss in the HRFZ-Si layer in the hybrid Tamm cavity, so that the permittivity of HRFZ-Si has an imaginary part. To study the

influence of the absorption loss of HRFZ-Si, we analyze its influence on the reflection spectrum of the cavity by changing the imaginary part of the refractive index of the HRFZ-Si, and here we focus on the reflection spectra at 0.40, 0.48, and 0.65 THz. The reflection spectra of the hybrid Tamm cavity calculated with TMM at $d_{substrate}$ = 510 μm are plotted in Fig. S10. The three resonance points are marked. The resonance frequencies of the hybrid Tamm cavity hardly changed for different values of the imaginary part, but the amplitude changed significantly. Notably, compared with the real part of the refractive index of the HRFZ-Si layer, the imaginary part of silicon has a greater influence on the amplitude of the reflection coefficient. The Q of the hybrid Tamm cavity is adversely affected by an increased imaginary part of silicon, particularly within the substrate layer. Notably, when employing an imaginary part of silicon (k_{Si}) value of 0.0008, the measured Q closely aligns with the theoretical Q calculated using TMM.

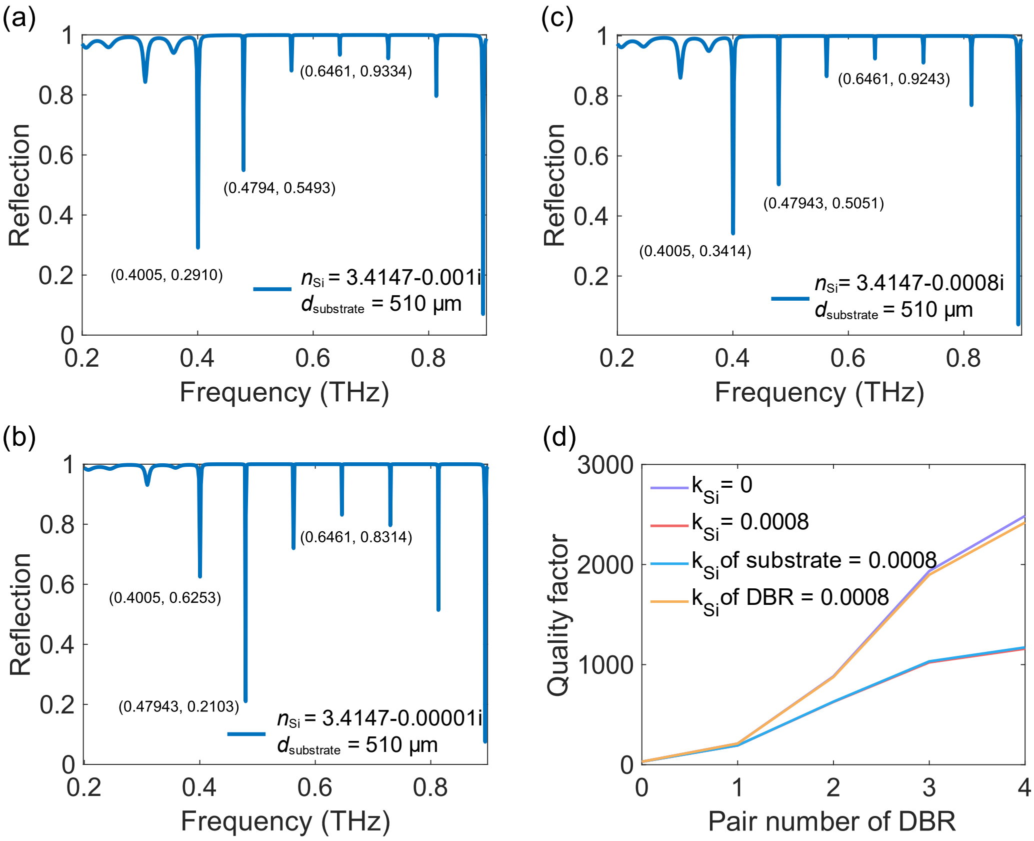

Fig. S10. Calculated reflection spectra for different imaginary parts of the refractive index of the silicon layer at $d_{substrate}$ = 510 μm: (a) n_{Si} = 3.4147 - 0.001i, (b) n_{Si}= 3.4147 -0.0008i, (c) n_{Si} = 3.4147-0.00001i. (d) Calculated Q values at a resonant mode (0.48 THz) of the detector with different numbers of layers with k_{Si} = 0, k_{Si} = 0.0008, k_{Si} of the substrate layer = 0.0008 and k_{Si} of the DBR = 0.0008.
